# Defining Disease Heterogeneity to Guide the Empirical Treatment of Febrile Illness in Resource Poor Settings

**DOI:** 10.1371/journal.pone.0044545

**Published:** 2012-09-21

**Authors:** Lisa J. White, Paul N. Newton, Richard J. Maude, Wirichada Pan-ngum, Jessica R. Fried, Mayfong Mayxay, Rapeephan R. Maude, Nicholas P. J. Day

**Affiliations:** 1 Centre for Clinical Vaccinology and Tropical Medicine, Nuffield Department of Clinical Medicine, Churchill Hospital, University of Oxford, Oxford, United Kingdom; 2 Faculty of Tropical Medicine, Mahidol University, Bangkok, Thailand; 3 Wellcome Trust-Mahosot Hospital-Oxford Tropical Medicine Research Collaboration, Microbiology Laboratory, Mahosot Hospital, Vientiane, Lao People's Democratic Republic; 4 Faculty of Postgraduate Studies, University of Health Sciences, Vientiane, Lao People's Democratic Republic; London School of Hygiene and Tropical Medicine, United Kingdom

## Abstract

**Background:**

Malaria incidence is in decline in many parts of SE Asia leading to a decreasing proportion of febrile illness that is attributable to malaria. However in the absence of rapid, affordable and accurate diagnostic tests, the non-malaria causes of these illnesses cannot be reliably identified. Studies on the aetiology of febrile illness have indicated that the causes are likely to vary by geographical location within countries (i.e. be spatially heterogeneous) and that national empirical treatment policies based on the aetiology measured in a single location could lead to inappropriate treatment.

**Methods:**

Using data from Vientiane as a reference for the incidence of major febrile illnesses in the Lao People's Democratic Republic (Laos) and estimated incidences, plausible incidence in other Lao provinces were generated using a mathematical model for a range of national and local scale variations. For a range of treatment protocols, the mean number of appropriate treatments was predicted and the potential impact of a spatially explicit national empirical treatment protocol assessed.

**Findings:**

The model predicted a negative correlation between number of appropriate treatments and the level of spatial heterogeneity. A spatially explicit national treatment protocol was predicted to increase the number of appropriate treatments by 50% for intermediate levels of spatial heterogeneity.

**Conclusions:**

The results suggest that given even only moderate spatial variation, a spatially explicit treatment algorithm will result in a significant improvement in the outcome of undifferentiated fevers in Laos and other similar resource poor settings.

## Introduction

Paradoxically, as malaria incidence declines in parts of SE Asia, an increasingly recognised important problem is how to treat patients presenting with non-malaria febrile illness. A wide range of infectious agents such as dengue, scrub typhus, murine typhus, typhoid and leptospirosis cause fevers, which often cannot be reliably distinguished from malaria in the absence of rapid, affordable and accurate diagnostic tests. Where the cause of particular non-malaria febrile illness is not identified using diagnostic tests, an empirical treatment protocol is deployed. That is, the infection is treated based on its most probable cause. The assumption about the most probable cause can be based on epidemiological information from the geographical setting of the infection.

Studies of specific patient groups (for example children and those with HIV or pneumonia) have described the causes of fever and proposed and tested empirical treatment guidelines [1], [2], [3]. Laboratory confirmation of the causes of fever is not necessarily carried out in either resource poor [4] or rich [5] settings. In a study in France, the national empirical treatment protocol for the treatment of respiratory infections (published by *Société de Pathologie Infectieuse de Langue Française* [6]) was demonstrated to be appropriate [5], whereas in rural India misdiagnosis of malaria was common, leading to frequent inappropriate treatments (i.e. 40% of non-malarial acute undifferentiated fever cases testing negative for malaria by RDT were treated with antimalarials) [4]. Spatial heterogeneity in the aetiology of febrile illness has been demonstrated at a local level [7]. Even if the aetiologies of febrile illness are known for a location, it is not necessarily appropriate to apply this information to a whole nation.

The Lao People's Democratic Republic (Laos) is a land-linked country with considerable ethnic and geographical variation. *Falciparum* malaria remains a public health problem but has declined in the last decade to a heterogeneous distribution with much higher incidences in the south [8]. There has been limited medical research but recent evidence, obtained to inform health policy, suggests that typhoid and other causes of community-acquired septicaemia, scrub typhus, murine typhus, dengue and leptospirosis are important causes of undifferentiated fever which are difficult to distinguish from malaria [9], [10]. However, most of these data were collected in the capital city, Vientiane [11], which, although small and including rural villages, is a very different environment from rural Laos where 83% of the population live [12]. Accessible laboratory diagnosis for the main causes of non-malaria fever is currently only available in Vientiane. Treatment of non-malaria febrile patients in the rest of the country is based on local clinical experience or protocols from Vientiane. The increased availability of malaria rapid diagnostic tests has demonstrated that much of what is diagnosed clinically as malaria is not [13].

Laos does not currently have a national empirical treatment protocol for non-malaria febrile patients. Since the only published data available are from Vientiane, the design of a national empirical treatment protocol would logically be based on the relative causes of non-malaria fever in Vientiane. However, given the ethnic and geographical diversity, it is likely that the aetiology of fever in different areas of rural Laos differs from that recorded in the capital, and thus national empirical treatment protocols based on those used in the capital may not be effective. Unpublished data from studies to determine the incidence fever at two diverse sentinel areas in rural Laos support this assertion by showing, for example, that typhoid was more common in northern Laos and dengue more common in the south [14]. Such regional diversity in the aetiology of fever may necessitate spatially-explicit empirical treatment protocols.

Since, like most developing countries, spatially explicit empirical data is not routinely collected a mathematical model was developed to explore the potential impact of different region-specific empirical treatment algorithms versus a single country-wide algorithm (based on Vientiane data) for Lao adults with undifferentiated fever.

## Methods

### The data

**Figure 1:**
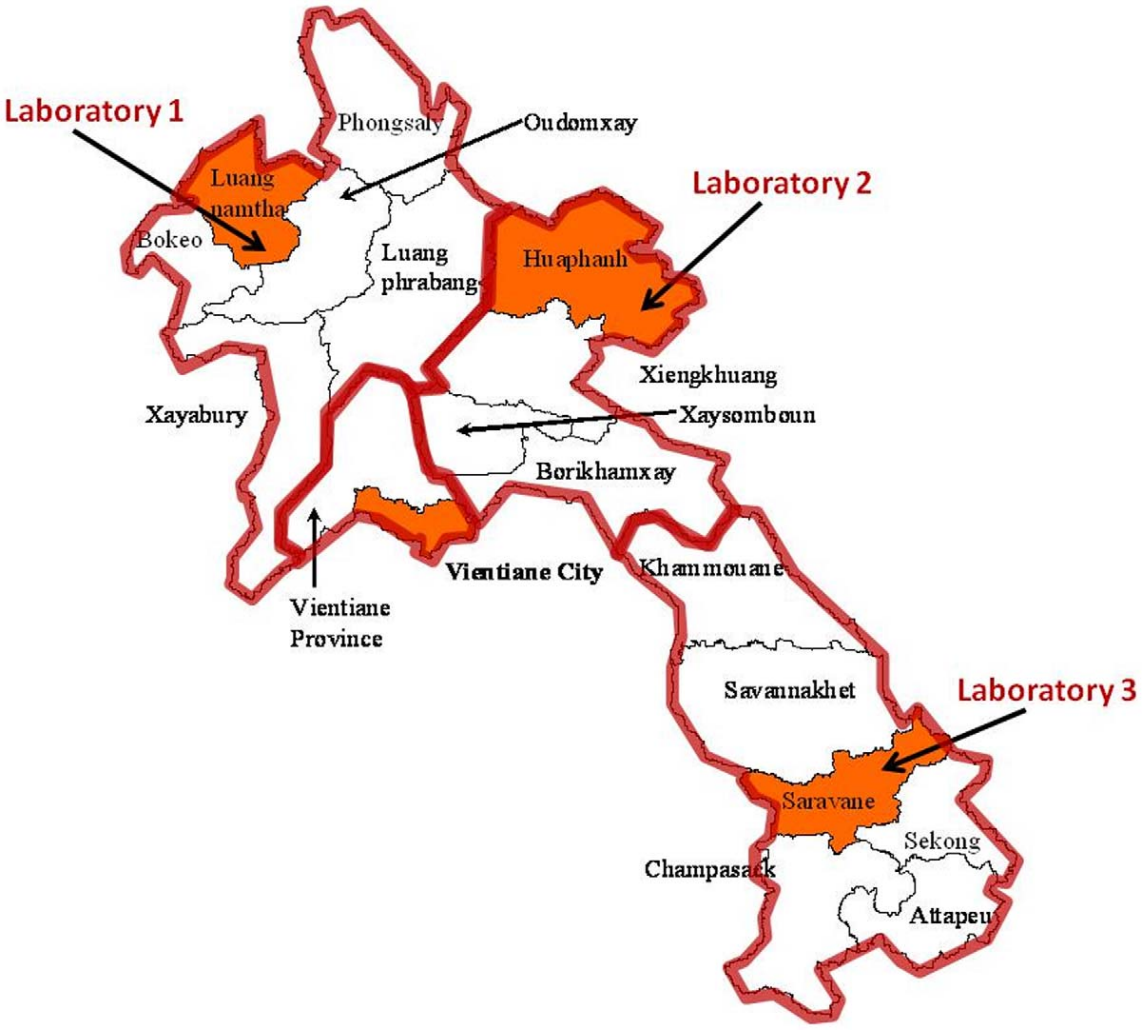
A map of Laos showing provinces. Vientiane and the provinces suggested for sentinel laboratories 1, 2 and 3 are marked in orange.

The following data were used as inputs in the model:

- Estimated incidence of febrile diseases, Mahosot Hospital, Vientiane (Table 1) [9], [10]
- Adult population sizes in each province (Table 2) [15]
- Positions of the three current diagnostic laboratories (Figure 1)
- Estimated proportion of febrile individuals who seek treatment in rural areas compared with Vientiane (0.5)
- The three sentinel provinces to be included in new treatment protocols plus Vientiane City & province (Figure 1)
- For each treatment and each disease, the estimated percentage beneficial effect (these are the cure rates for drug treatments) for each infection given individual empirical treatments. (Table 3)
- For each individual empirical treatment, a threshold based on a combination of the proportional contribution to all-cause fever of its target infections and the treatment effect against those infections. In the areas of the country covered by each of Vientiane and the three provinces with laboratories, the individual treatment would be included in the protocol for values above this threshold. (Table 3)

**Table 1:** Estimated incidence and empirical treatment for the most common causes of fever in Vientiane.

Infection	Approximate adult annual incidence at Mahosot Hospital, Vientiane	Inferred incidence (capita^−1^ year^−1^) in Vientiane adults	Empirical treatment
Scrub typhus	70	0.00046	doxycycline
Murine typhus	60	0.00040	doxycycline
Typhoid	40	0.00026	ofloxacin
Dengue	30	0.00020	fluid balance management
Leptospirosis	30	0.00020	doxycycline
Pulmonary tuberculosis	25	0.00017	combination therapy
*E. coli* septicaemia	20	0.00013	ceftriaxone
*Staphylococcus aureus* septicaemia	15	0.00010	cloxacillin
*Klebsiella* spp. septicaemia	15	0.00010	ceftriaxone
*Burkolderia pseudomallei* (melioidosis)	30	0.00020	ceftazidine
*Streptococcus pneumoniae* septicaemia or meningitis	20	0.00013	ceftriaxone

**Table 2:** Adult populations of each Lao province in 2005 [15].

Province	Adult population
Phongsali	96,716
Louangnamtha	88,304
Bokeo	87,501
Oudomxai	149,283
Louangphrabang	237,094
Houaphan	152,820
Xaignabouri	214,885
Xiangkhoang	127,226
Viangchan	243,192
Vientiane City	503,961
Bolikhamxai	130,907
Khammouan	196,446
Savannakhet	497,246
Saravan	181,931
Xekong	46,430
Champasak	438,636
Attapu	63,637

**Table 3:** Estimated percentage beneficial effect on each infection of individual empirical treatments.

	doxycyline	ofloxacin	fluid balance management	combination therapy	ceftriaxone	cloxacillin	ceftazidine
Scrub typhus	85	0	0	0	0	0	0
Murine typhus	85	0	0	0	0	0	0
Typhoid	0	85	0	0	90	0	0
Dengue	0	0	70	0	0	0	0
Leptospirosis	85	0	0	0	80	0	0
Tuberculosis	0	0	0	80	0	0	0
*E. coli* septicaemia	0	70	0	0	85	0	0
*Staphylococcus aureus* septicaemia	0	0	0	0	85	85	0
*Klebsiella* spp. Septicaemia	0	70	0	0	85	0	0
Melioidosis	0	0	0	0	0	0	90
Pneumococcal septicaemia or meningitis	0	0	0	0	100	0	0
Inclusion threshold	25	20	20	20	40	20	40

Inclusion threshold: percentage of all febrile cases of diseases treatable by each individual empirical treatment that must be exceeded for the inclusion of that treatment in an empirical protocol.

### The model

The mathematical model is a static model that generates a set of possible incidences of fever in each Lao province, based on the incidence in Vientiane and a range of variation compared with the Vientiane incidence, as described below.

The map of Laos is divided into four regions: Vientiane, North, Central and South (illustrated by the red boundaries in Figure 1). Each region has a sentinel province (illustrated by orange shading in Figure 1) from which approximate incidence data are either already available (in the case of Vientiane) or estimated.

Two levels of variation are considered: national and provincial. For each province, a set of random incidences for each disease is generated, *inc_rand_*, based on the incidence of fever in Vientiane, the population size of the province and treatment seeking behaviour. Two formulations for generating a random incidence were used. First, the list of incidences for Vientiane was randomly re-assigned to the list of diseases (“shuffling"). Secondly, for each disease, a value for the incidence was sampled from a normal distribution with a mean equal to the mean of the 11 disease incidences of Vientiane and a standard deviation equal to the mean (“sampling").

Then, for the sentinel provinces (Figure 1), for each level of national variation, *h_N_*, a new incidence, *inc_N_*, for each disease is generated from the equation:
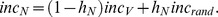
The value of *h_N_* is between zero and unity. A value of zero represents a perfect correlation between the incidence of the 11 diseases in the sentinel provinces and those in Vientiane, whereas a value of unity indicates no correlation between Vientiane and the sentinel provinces.

For the remaining provinces (Figure 1), , for each level of provincial variation, *h_P_*, a new incidence for each disease, *inc_P_*, is generated from the incidence in the sentinel province, *inc_N_*, using the equation:
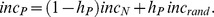
The value of *h_P_* is between zero and unity. A value of zero represents a perfect correlation between the incidence of the 11 diseases in the remaining provinces and those in in their sentinel province, whereas a value of unity indicates no correlation between the sentinel provinces and their surrounding provinces.

The algorithm for the design of a treatment protocol based on a given set of incidences is as follows.

- For each treatment and each febrile illness, multiply the incidence of the illness by the effect of the treatment on that illness.
  1. For example for doxycycline and murine typhus in Vientiane (see Tables 1 and 3) this value would be 85% of 0.0004
- Then take the sum of all these values for the given treatment.
  1. For example for doxycycline in Vientiane the sum would be 0.0009.
- Then express this value as a percentage of the sum of the incidence of all febrile illness
  1. For example, for doxycycline in Vientiane this percentage would be 38%.
- Then if this percentage is greater than or equal to the treatment threshold then the treatment is included in the protocol
  1. For example for Vientiane the percentages are for doxycycline, ofloxacin, fluid balance management, combination therapy, ceftriaxone, cloxacillin and ceftazidinecefazidine are 38%, 16%, 6%, 6%, 34%, 4% and 8% respectively. The thresholds are given in Table 3 as 25%, 20%, 20%, 20%, 40%, 20% and 40%. Therefore an empirical treatment protocol based on the epidemiology of Vientiane, assuming the treatment effects and thresholds listed in Table 3 would be doxycycline alone.

This algorithm allows different empirical treatment protocols to be designed automatically for different sets of incidences. For example, if we assume that the aetiology in another hypothetical province has no correlation with that in Vientiane, a randomly generated set of incidences for [Scrub typhus, Murine typhus, Typhoid, Dengue, Leptospirosis, Tuberculosis, *E. coli* septicaemia, *Staphylococcus aureus* septicaemia, *Klebsiella* spp. septicaemia, melioidosis and Pneumococcal septicaemia or meningitis] sampled from a normal distribution with a mean and standard deviation equal to the mean incidence of the febrile illnesses in Vientiane could be [0.00086, 0.00037, 0.00020, 0.00037, 0.00017, 0.00019, 0.00053, 0.00051, 0.00051, 0.00036, 0]. Applying the above algorithm would result in an empirical treatment protocol of doxycycline, ofloxacin and ceftriaxone.

For each pair of national and provincial variations, (*h_N_*,*h_P_*), a set of potential incidences was generated for febrile diseases in each province. For each level of spatial variation, the number of patients that would be appropriately treated if a protocol based on the aetiology in Vientiane was predicted. The number of patients that would be appropriately treated if a spatially explicit protocol were applied to each region (based on the epidemiology of its sentinel province and the inclusion thresholds (Table 3)) was also calculated. The difference between these two values, summed over all diseases and all provinces, is defined as the potential impact of a spatially explicit treatment protocol for fever in Laos.

## Results

The model was run 10,000 times for each pair of variations (national and provincial).

**Figure 2:**
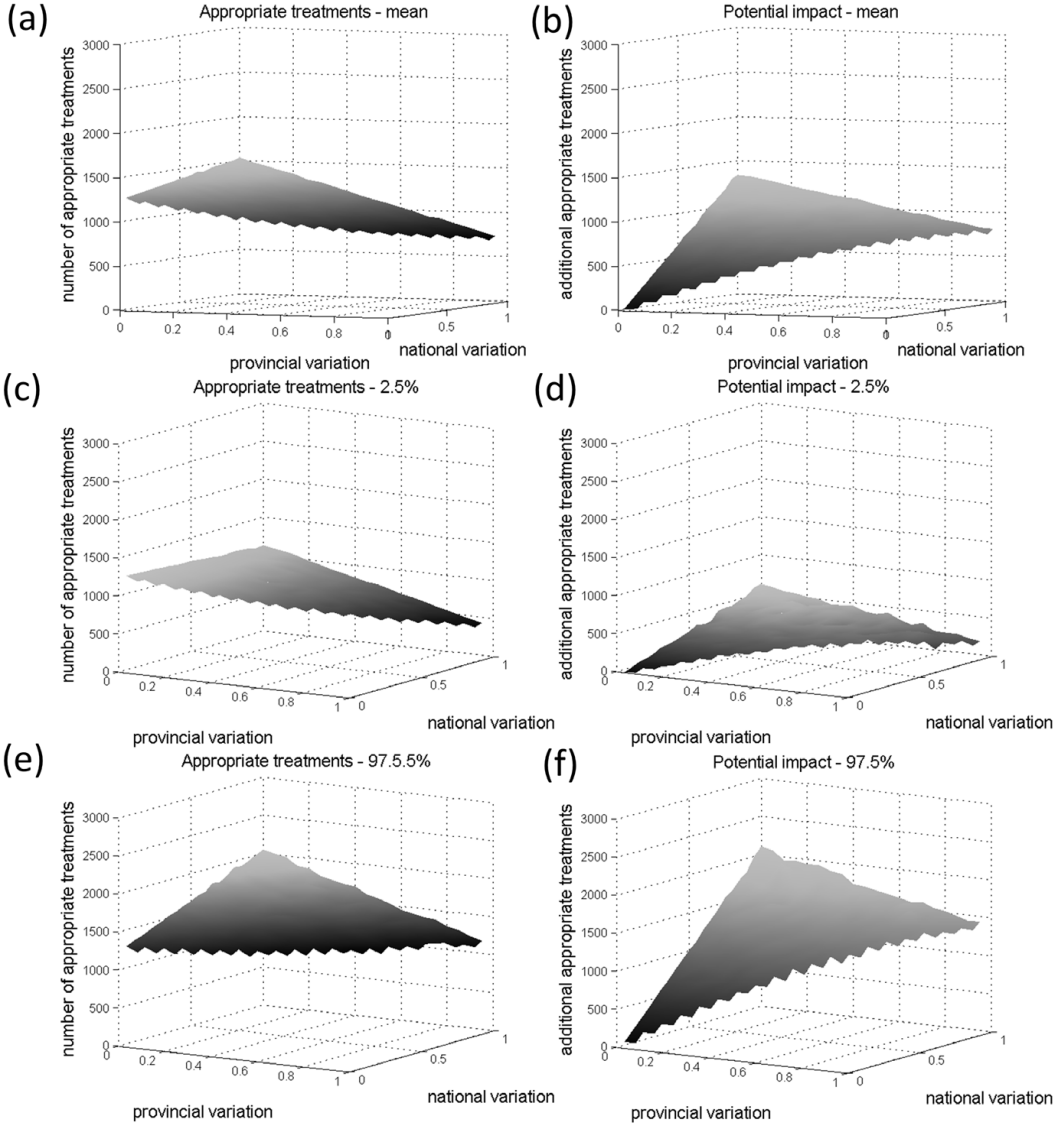
Graphs illustrating the model predictions of the effect of spatial heterogeneity given the baseline input values in Tables (1, 2, 3). The mean predicted numbers of appropriate treatments if a protocol based on the Vientiane epidemiology is applied nationally is plotted in Graph (a) for a range of values for national and regional variation. The 2.5% and 97.5% prediction intervals are plotted in Graphs (c) and (e). The mean additional numbers of appropriate treatments (i.e. the potential impact) predicted if a spatially explicit treatment protocol based on the incidence not only in Vientiane but also in three sentinel provinces were applied is plotted in Graph (b) for the same range of national and regional variation. The 2.5% and 97.5% prediction intervals are plotted in Graphs (c) and (f).

Provincial variation had a greater effect on the predicted number of appropriate treatments than national variation. In general, a negative correlation between number of appropriate treatments and the level of spatial heterogeneity was predicted (Figure 2, left). The potential impact of a spatially explicit treatment protocol (with specific sub-protocols for Vientiane, Northern, Central and Southern provinces) is positively correlated with the level of national spatial variation.

Provincial variation acts mainly to increase the uncertainty about the potential impact, with the confidence intervals becoming wider with increasing spatial heterogeneity at the provincial level. The potential impact is in the order of ∼1000 (0 to 2000) additional appropriate treatments/year for intermediate levels of spatial heterogeneity, which represents about 12% (0% to 25%) of the predicted underlying annual incidence, that is an additional 50% (0% to 100%) of the predicted number of appropriate treatments under the current protocol.

A sensitivity analysis (see Figure S1) was performed varying all the values used in the model within realistic ranges. This analysis confirmed that the results presented are qualitatively robust and that the highest impacts are predicted for the greatest spatial heterogeneity. The results are robust to the choice of randomization method. The analysis also shows that the higher the percentage of febrile patients who seek treatment outside the capital and the greater the therapeutic effect of the treatments, the higher the predicted number of appropriate treatments and potential impact. The potential impact is very sensitive to the threshold conditions on incidence for inclusion of medicines in the national and regional protocols. Low thresholds can lead to predictions of low and even negative impact since, for low thresholds the pre-existing protocol is already likely to be optimised for the nation due to its broad spectrum. A threshold ∼0.9 of the baseline predicts the highest impact.

## Discussion

The challenge to recommend a national empirical treatment protocol that is both appropriate given the local epidemiology and parsimonious, since cost and availability will be limiting factors, has been considered using a mathematical model. If treatment protocols are designed to comprise up to two combined treatments per province, the model predicts that a tailored spatially explicit protocol designed using field data would have a high impact on the numbers of appropriately treated cases. The potential impact is estimated to be in the order of c. 1000 additional appropriate treatments/year for adults in Laos (an increase of 50%). This hypothesis could be tested by conducting clinical epidemiological studies to determine the causes of undifferentiated fever at diverse ‘sentinel’ study sites, with appropriate microbiology laboratory and clinical support.

Despite Laos being a country of small land area (∼ the size of the UK) there are quite marked geographical differences in the aetiology of fevers. In a study of the aetiology of fever amongst those with negative malaria rapid diagnostic tests, dengue and malaria were more frequent in the south and leptospirosis and typhoid more frequent in the north [14].

Geographical and temporal heterogeneity of antimicrobial resistance patterns will also have an important impact. Currently multidrug resistant typhoid and methicillin resistant *S. aureus* are rare but extended spectrum B lactamase (ESBL) bacteria relatively common [9], [16]. How these resistance patterns change will profoundly influence the efficacy of empirical treatment of these pathogens.

With increased focus on the causes and treatment of undifferentiated fever, it is vital that spatial diversity in aetiology of fever, and hence treatment protocols are well defined. Further, more studies are needed to test this hypothesis and define the degree of heterogeneity and its environmental and human determinants. Without this, we risk implementing inappropriate empirical treatment protocols and not fulfilling the public health promise that the control of non-malarial fevers holds in resource poor settings.

## Supporting Information

Figure S1 **The results of a sensitivity analysis for a set of pairs of national and provincial variation, (** ***h_N_*** **,** ***h_P_*** **) = {(0.2,0.2), (0.5,0.2), (0.8,0.2), (0.5,0.5), (0.8,0.5), (0.8,0.8)}, in the form of 4 panes for each pair with an array of graphs (values given in the title of each pane).** The panes represent: mean number of appropriate treatments (top left); mean potential impact (bottom left); mean number of treatments included in provincial protocols (top right); mean number of either ceftriaxone or ceftazidime included in provincial protocols (bottom right). The threshold condition for inclusion of the drugs in a treatment protocol as a multiple of the baseline set of conditions for ratios of 0.1 to 1.2 on the x-axis of each graph. Treatment seeking behaviour levels for rural residents for ratios of 0.3, 0.5 and 1 compared with Vientiane (increasing values along the rows). Treatment effects for ratios of 0.3, 0.5 and 1 compared with baseline levels (increasing values up the columns). The three choices for randomization of shuffling, normal distribution with standard deviation equal to 0.5 of the mean, normal distribution with standard deviation equal to the mean (black blue and red lines respectively).
(DOC)
