# Supplementary figures and images for: Defining Disease Heterogeneity to Guide the Empirical Treatment of Febrile Illness in Resource Poor Settings
Source: PLoS One. 2012 Sep 21;7(9):e44545. doi: 10.1371/journal.pone.0044545 (PMC3448597; doi:10.1371/journal.pone.0044545)

**Figure S1**


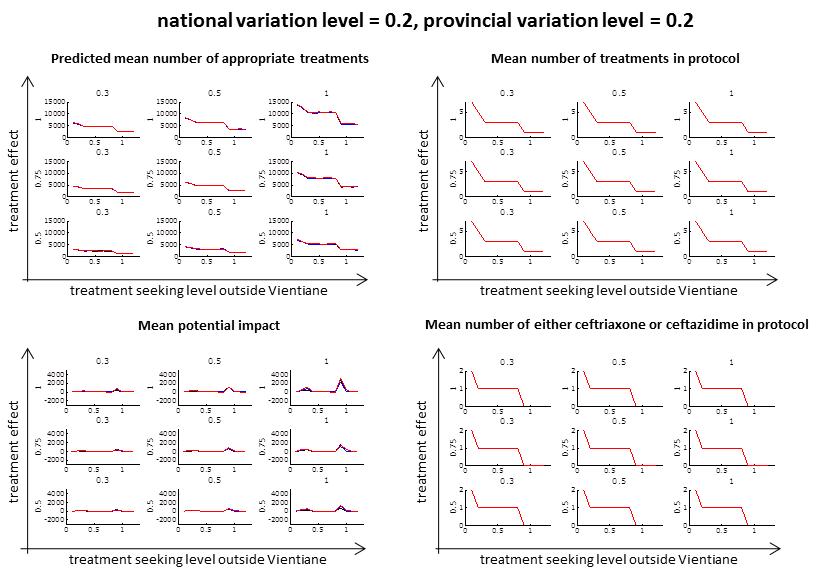


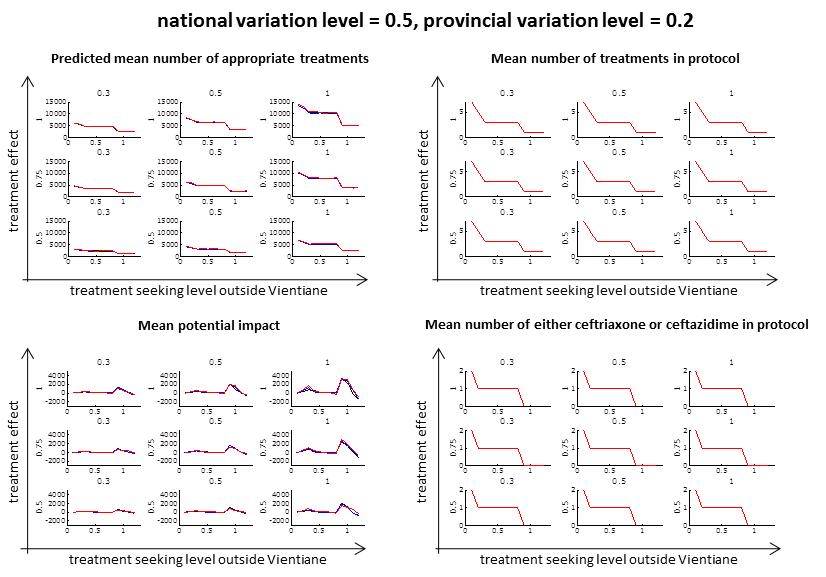


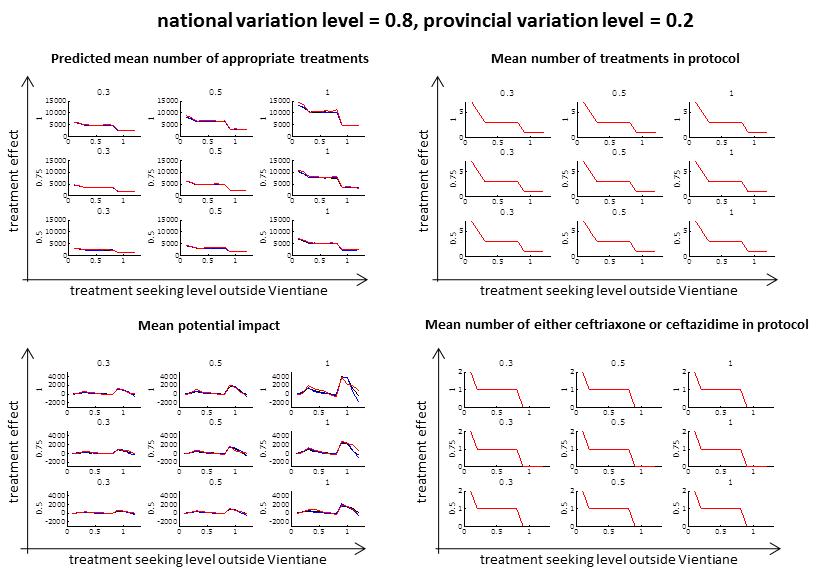


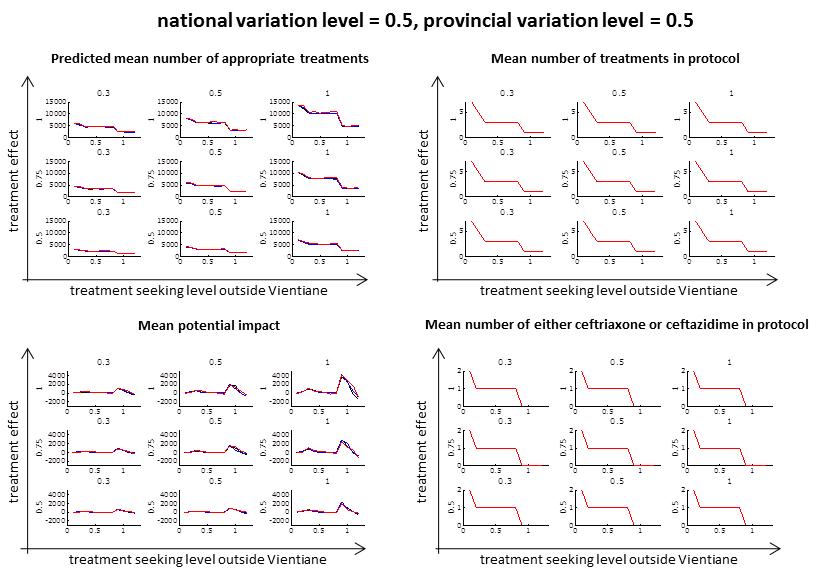


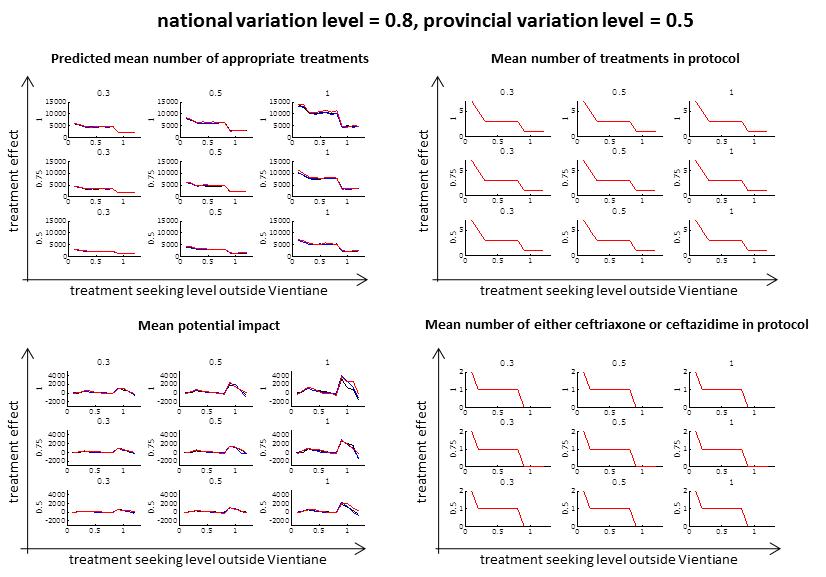


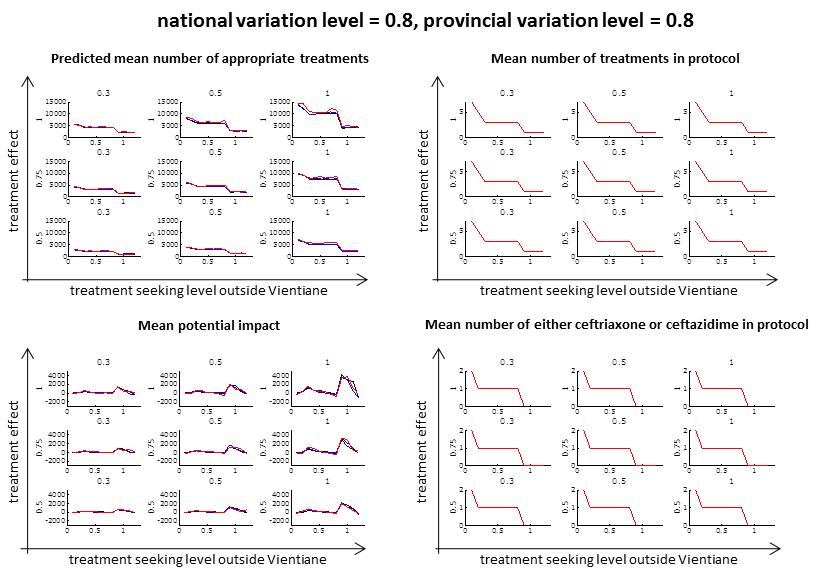

Supplement: Figure S1 — The results of a sensitivity analysis for a set of pairs of national and provincial variation, ( hN , hP ) = {(0.2,0.2), (0.5,0.2), (0.8,0.2), (0.5,0.5), (0.8,0.5), (0.8,0.8)}, in the form of 4 panes for each pair with an array of graphs (values given in the title of each pane). The panes represent: mean number of appropriate treatments (top left); mean potential impact (bottom left); mean number of treatments included in provincial protocols (top right); mean number of either ceftriaxone or ceftazidime included in provincial protocols (bottom right). The threshold condition for inclusion of the drugs in a treatment protocol as a multiple of the baseline set of conditions for ratios of 0.1 to 1.2 on the x-axis of each graph. Treatment seeking behaviour levels for rural residents for ratios of 0.3, 0.5 and 1 compared with Vientiane (increasing values along the rows). Treatment effects for ratios of 0.3, 0.5 and 1 compared with baseline levels (increasing values up the columns). The three choices for randomization of shuffling, normal distribution with standard deviation equal to 0.5 of the mean, normal distribution with standard deviation equal to the mean (black blue and red lines respectively). (DOC) [file pone.0044545.s001.doc]
